# Tislelizumab-induced myositis antibody-negative myasthenia gravis in patients with urothelial carcinoma: a case report

**DOI:** 10.3389/fimmu.2026.1854332

**Published:** 2026-07-01

**Authors:** Yi Bu, Hongmei Wang, Guangmei Jiao, Shuo Zhang, Xiaoxuan Zhang

**Affiliations:** 1 Department of Neurology, affiliated Hospital of Chengde Medical University, Chengde, Hebei, China; 2 Hebei Key Laboratory of Panvascular Diseases, Chengde, Hebei, China

**Keywords:** adverse reactions, antibody-negative myasthenia gravis, myositis, tislelizumab, urothelial carcinoma

## Abstract

Tislelizumab, an immune checkpoint inhibitor, is widely used in the treatment of various malignancies; however, its immune-related adverse events(irAEs) require careful attention. Here, we report a case of a patient with recurrent urothelial carcinoma who developed myositis and myasthenia-like manifestations after receiving tislelizumab, despite negative myasthenia-associated antibodies. The patient’s symptoms gradually alleviated following treatment with corticosteroids and intravenous immunoglobulin. To our knowledge, antibody-negative myasthenia gravis associated with tislelizumab has been scarcely documented. This case highlights that in patients receiving tislelizumab, early pharmacological intervention should be considered when typical myasthenia-like manifestations occur, even in the absence of antibody positivity, emphasizing the importance of prompt recognition and timely treatment.

## Introduction

Immune checkpoint inhibitors (ICIs) are a class of agents that restore or enhance antitumor immune responses by blocking immune checkpoint pathways and are widely used in cancer therapy (1). Based on their targets, ICIs are mainly classified into inhibitors of cytotoxic T-lymphocyte-associated antigen 4 (CTLA-4), programmed cell death receptor 1 (PD-1), programmed death-ligand 1 (PD-L1), and bispecific antibodies (1, 2). However, with their increasing clinical use, irAEs have gained growing attention, including neurological complications involving both the central and peripheral nervous systems. These manifestations include encephalitis, myelitis, neuromuscular junction disorders, peripheral neuropathy, and myopathy (3, 4).

Tislelizumab, a PD-1 inhibitor, has been reported to cause myasthenia gravis (5). In the present case, the patient developed myositis and myasthenia-like manifestations after receiving tislelizumab. The symptoms gradually improved following treatment with corticosteroids and intravenous immunoglobulins (IVIG). Notably, repeated tests for myasthenia gravis–related antibodies were negative, which posed a diagnostic challenge. This case may contribute to a better understanding of tislelizumab-related adverse reactions and serve as a reference for early recognition and management of immune-related neurological complications.

## Case presentation

**Figure 1:**
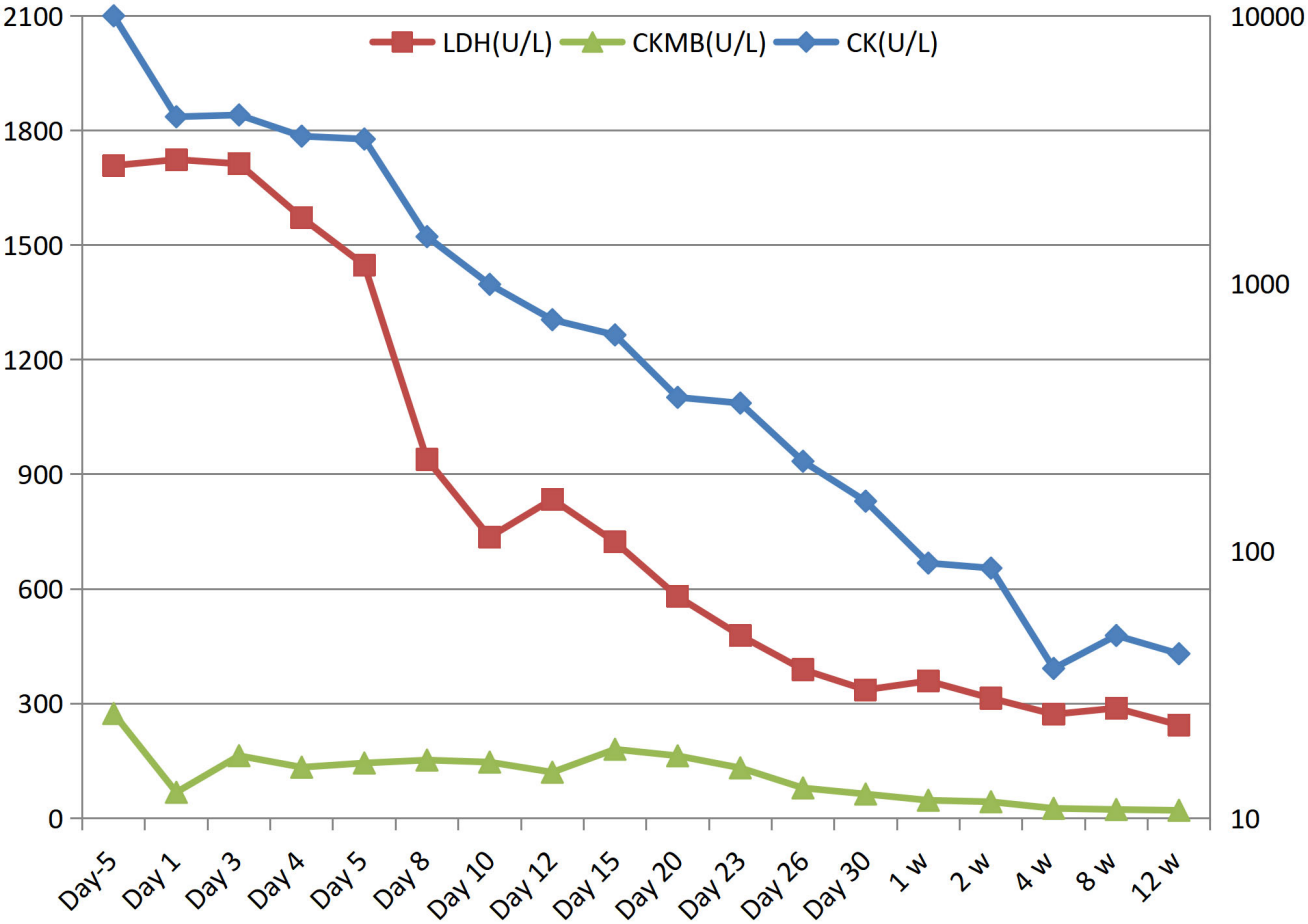
Changes to the patient’s cardiac markers. LDH, Lactate Dehydrogenase; CKMB, Creatine Kinase MB; CK, Creatine Kinase.

A 75-year-old man was admitted to our hospital with a 5-day history of bilateral lower limb weakness, pain, ptosis, and dyspnea. The symptoms exhibited a typical fluctuation pattern: mild in the morning, worsening in the evening, with slight improvement after rest. Outpatient examinations, including pulmonary CT, electrocardiogram (ECG), brain natriuretic peptide (BNP), and troponin I, revealed no abnormalities. However, serum creatine kinase (CK) was markedly elevated (>10,000 U/L) (**Figure 1**). Immune-mediated myositis was initially suspected. After treatment with prednisone sodium succinate (105mg), the CK level decreased to 6516 U/L, but limb pain and weakness showed no significant improvement.

Three years previously, the patient had been diagnosed with urothelial carcinoma with a clinical stage of cT1N0M0. He underwent laser enucleation followed by regular intravesical instillation of doxorubicin hydrochloride. One year prior to admission, cystoscopy revealed tumor recurrence, and the same treatment regimen was repeated. One month before admission, cystoscopy again confirmed tumor recurrence. Seventeen days prior to admission, the patient received combination therapy with tislelizumab (200 mg i.v. every 3 weeks) plus 21 days cycles of gemcitabine (1000 mg/m^2^ i.v. on days 1 and 8 of each cycle). Upon the onset of symptoms, the patient discontinued the aforementioned treatment regimen.

**Figure 2:**
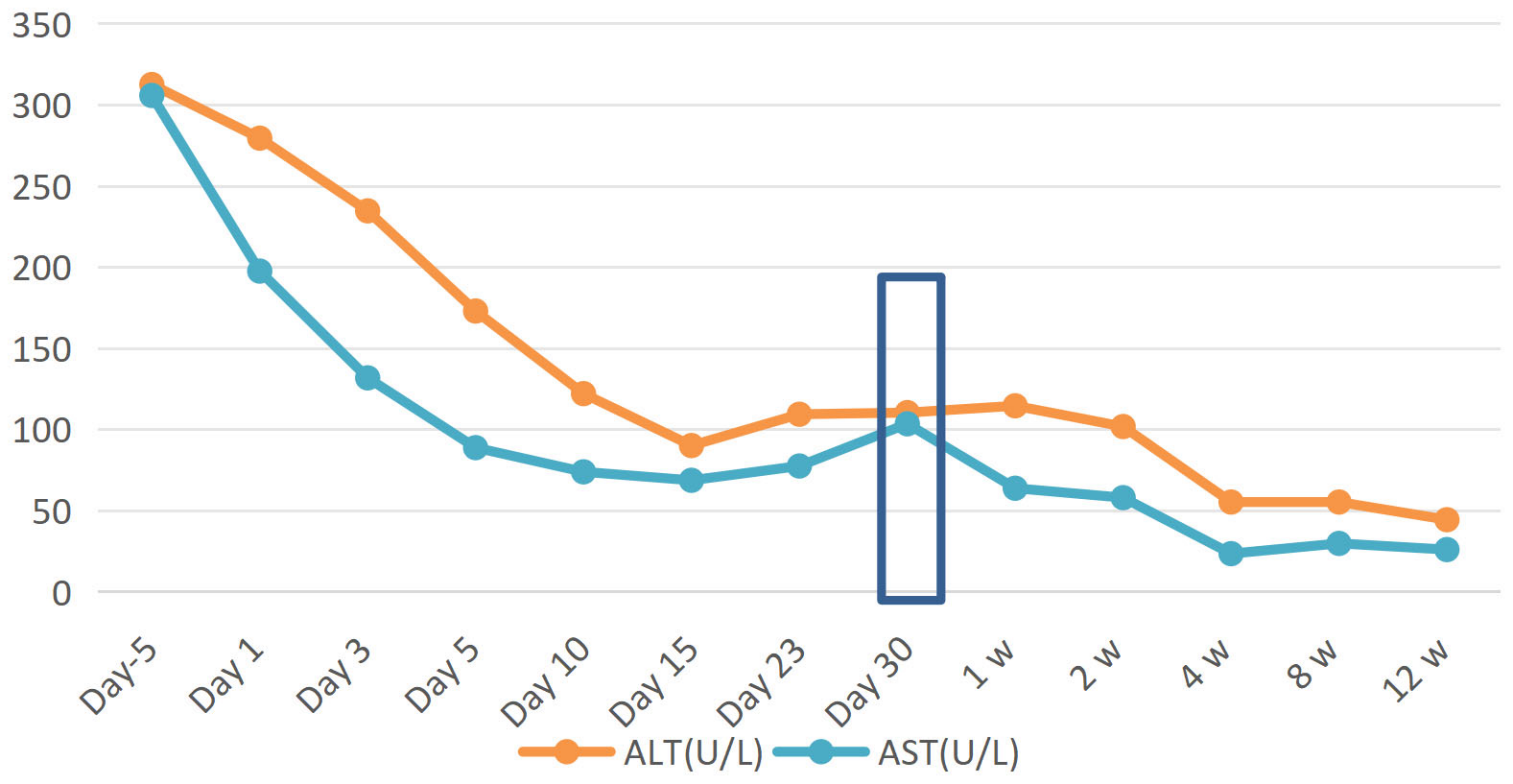
Changes to the patient’s liver function indexes. ALT,alanine aminotransferase; AST, aspartate aminotransferase.

**Figure 3:**
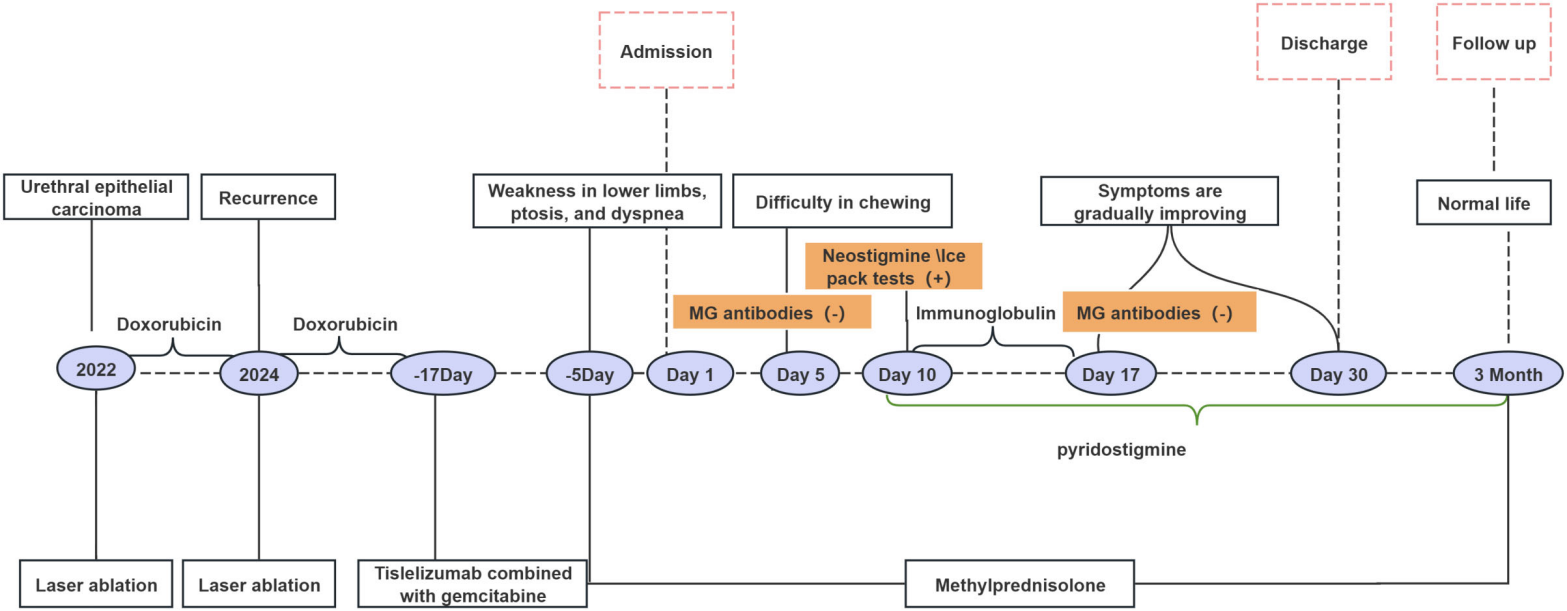
Symptoms and interventions of patients before and after treatment.

On admission, the patient presented with bilateral ptosis, with a vertical palpebral fissure ratio of 2:7 between the left and right eyes. Tenderness and swelling were observed in the proximal muscles of both lower limbs, with muscle strength graded as 4/5. Laboratory tests revealed markedly elevated levels of CK, CKMB, ALT, and AST (**Figures 1**, **2**), suggesting immune checkpoint inhibitor-associated myositis with myasthenia-like manifestations. Intravenous methylprednisolone (65 mg once daily) was administered for immunosuppression. Further laboratory investigations showed negative results for paraneoplastic antibodies, myositis-specific antibodies, antinuclear antibodies, myasthenia gravis–related antibodies,including AChR、Musk、LRP4、Titin、RyR、SOX1 and Agrin(Cell-Based Assay), systemic vasculitis markers, and thyroid function tests. Lymphocyte subset analysis showed a CD3+CD4+/CD3+CD8+ ratio of 0.47.Due to the patient’s dyspnea, a complete muscle and cardiac MRI examination could not be performed, and the patient declined a muscle biopsy. Repetitive nerve stimulation showed no abnormalities, while needle electromyography suggested myogenic damage. Serial follow-up tests of myocardial enzymes and liver and kidney function demonstrated a gradual decline in serum CK, CK-MB, ALT, and AST levels; however, the patient’s clinical symptoms showed no significant improvement. On the fifth day of hospitalization, the patient developed dysphagia, masticatory weakness, prolonged meal duration, and persistent dyspnea.Given that myasthenia gravis was suspected,both modified ice pack test and the neostigmine test was performed. The procedures were as follows (1): Modified ice pack test: The patient maintained upward gaze for 2 minutes, followed by immediate application of an ice pack (0–4°C) to the closed eyelids for 3 minutes. The margin reflex distance (MRD) was measured, with improvement 4mm(≥2 mm) considered significant.(2)Neostigmine test:After 30 minutes, the patient’s ptosis returned to its ice pack test state.A 1.0 mg dose of neostigmine mesylate and 0.5 mg of atropine were administered intramuscularly; the atropine was intended to counteract cholinergic adverse effects.The upper eyelid fatigue duration was monitored every 10 minutes, and a relative score was calculated. The patient achieved a score of 100% (>60%), indicating a positive outcome. Both the neostigmine test and the ice pack test were positive, suggesting antibody-negative myasthenia gravis (MG). According to the MGFA classification, the patient was classified as type IIIb, with a QMG score of 18. Treatment with oral pyridostigmine (60 mg three times daily) resulted in significant improvement in dysphagia, with the QMG score decreasing to 16. On day 10 after admission, IVIGtherapy was initiated at a dose of 0.4 g/kg/day for 5 consecutive days. Following treatment, the patient’s ptosis, limb weakness, and dyspnea gradually improved, and masticatory weakness was alleviated, with the QMG score decreasing to 7. On day 17 of hospitalization, repeat testing for myasthenia gravis-related antibodies remained negative. On day 30, the patient was discharged with a prescribed for oral methylprednisolone (50 mg once daily), with a planned taper of 10 mg every 2 weeks. After maintaining a dose of 10 mg daily for one month, the medication was gradually discontinued. Pyridostigmine was also tapered and eventually discontinued. At follow-up, the patient’s symptoms of ptosis, limb weakness, and dyspnea had completely resolved, and normal eating and ambulation were restored.A cystoscopic follow-up examination was performed three months after discharge, showing no tumor recurrence (**Figure 3**).

## Discussion

In this case, the patient developed limb weakness and dyspnea approximately 3 weeks after the administration of tislelizumab therapy, which is consistent with the typical onset time of immune checkpoint inhibitor-related toxicity (6). According to the Common Terminology Criteria for Adverse Events (CTCAE, 5.0) established by the National Cancer Institute (NCI) of the U.S. National Institutes of Health (NIH), the patient was classified as having grade G2 myalgia and grade G3 myasthenia gravis. Following treatment with corticosteroids and IVIG, the patient showed a favorable response, with a rapid decline in muscle enzyme levels and gradual improvement in muscle weakness. These findings are consistent with the clinical outcomes reported in previous cases of adverse reactions associated with other immune checkpoint inhibitors (7, 8). These findings further support that the aforementioned manifestations were induced by tislelizumab. The patient presented with muscle pain, accompanied by serum muscle enzymes levels exceeding five times the upper limit of normal. Electromyography (EMG) demonstrated myogenic damage, suggesting skeletal muscles involvement. Unfortunately, the patient declined a muscle biopsy; therefore, pathological confirmation was not obtained. According to the *International Consensus on Immune Checkpoint Inhibitor–Associated Neurological Adverse Events (2021)*, the diagnosis of immune checkpoint inhibitor-induced myositis was considered reasonable. The patient exhibited elevated cardiac enzymes levels, warranting suspicion of myocarditis, however, he did not present with typical clinical manifestations of acute coronary syndrome (ACS), such as chest tightness or pain. Repeated re-examinations of brain natriuretic peptide (BNP) and troponin levels were within normal ranges, and echocardiography revealed no significant abnormalities. Multiple ECG examinations during hospitalization showed no evidence of myocardial ischemia. Given that the CK-MB/CK ratio was <0.06, the elevated cardiac enzymes levels were more likely attributable to skeletal muscle injury rather than myocardial damage (9). In addition, the patient showed elevated transaminase levels. However, there was no history of liver disease, and liver ultrasonography revealed no significant abnormalities. Combined with gamma-glutamyl transferase (GGT) levels within the normal range, the abnormal elevation of transaminases was considered likely secondary to skeletal muscle injury rather than hepatic dysfunction (10).

The patient presented with ptosis, dysphagia, and dyspnea with fluctuating symptoms. Both the neostigmine test and ice pack test were positive. After treatment with pyridostigmine, the patient’s symptoms improved significantly, which is consistent with the typical clinical features of myasthenia gravis. However, myasthenia gravis-related antibody tests were negative on two occasions, and electrophysiological studies showed no evidence of neuromuscular junction impairment, posing a diagnostic challenge. According to the *International Consensus on Immune Checkpoint Inhibitor–Associated Neurological Adverse Events (2021)*, the patient was diagnosed with antibody-negative myasthenia gravis.

Previous studies have shown that approximately 80%-85% of patients with primary myasthenia gravis are positive for ACHR antibodies, whereas the proportion of antibody-positive patients among those with immune checkpoint inhibitor-associated myasthenia gravis is only about 30% (11). The occurrence of antibody-negative myasthenia gravis induced by tislelizumab is scarcely documented in the literature. The possible reasons are as follows: First, tislelizumab may bypass acetylcholine receptors-mediated pathways and activate cytotoxic T cells, leading to injury of the neuromuscular junction (8). In the present case, the CD8/CD4 ratio of lymphocyte subsets was elevated, suggesting that T-cell activation may have contributed to the onset and progression of myasthenia gravis. Second, the complexity of immune checkpoint inhibitor-related overlapping syndromes should be considered. A retrospective study reported that immune checkpoint inhibitor-induced myositis and MG may coexist, and that some patients may present with histopathologic inflammation changes without detectable antibody production (12). Third, antibodies may appear later in the disease course, and early testing may yield false-negative results due to a limited detection window (13). In this case, the interval between the two antibody tests was only 7 days, and the possibility of false-negative results due to early testing cannot be ruled out.

## Conclusions

This case underscores that tislelizumab, despite its efficacy in treating malignancies such as urothelial carcinoma, can induce rare but severe neuromuscular irAEs, including antibody-negative myasthenia gravis and myositis. The absence of detectable myasthenia-associated antibodies should not preclude the diagnosis when clinical manifestations are suggestive, as timely intervention with corticosteroids and intravenous immunoglobulin may lead to significant symptom improvement. Clinicians should maintain a high index of suspicion for neuromuscular irAEs in patients receiving tislelizumab and initiate prompt immunomodulatory therapy to mitigate morbidity and potential life-threatening complications.

## Data Availability

The original contributions presented in the study are included in the article/Supplementary Material. Further inquiries can be directed to the corresponding author.
